# Infizierte, pathologische Humerusfraktur bei Sichelzellanämie – noch ein Einzelfall?

**DOI:** 10.1007/s00113-023-01334-9

**Published:** 2023-06-04

**Authors:** Tobias Malte Ballhause, Philip Linke, Annika Hättich, Till Orla Klatte, Karl-Heinz Frosch, Konrad Mader

**Affiliations:** 1 Klinik und Poliklinik für Unfallchirurgie und Orthopädie, Universitätsklinikum Hamburg-Eppendorf, Martinistr. 52, 22529 Hamburg, Deutschland; 2 Unfallchirurgie, Orthopädie und Sporttraumatologie, BG Klinikum Hamburg, Hamburg, Deutschland

**Keywords:** Sichelzellkrankheit, Osteomyelitis, Knochennekrose, Vasookklusive Krise, *Klebsiella aerogenes*, Sickle cell disease, Osteomyelitis, Bone necrosis, Vaso-occlusive crisis, *Klebsiella aerogenes*

## Abstract

Die vasookklusiven Krisen der Sichelzellkrankheit gehen mit Knochennekrosen einher, welche eine endogene Keimbesiedlung und somit eine Osteomyelitis begünstigen. Dies bringt eine große Herausforderung für die Eradikation und Frakturversorgung mit sich.

Ein 22-jähriger Patienten mit Sichelzellkrankheit erlitt eine mehrfragmentäre Humerusschaftfraktur. Im Rahmen der operativen Versorgung entleerte sich Pus im Frakturbereich, und es zeigte sich in der weiterführenden Diagnostik eine Osteomyelitis mit dem Nachweis von *Klebsiella aerogenes*. Fünf Monate vor dem Unfall kam es zu einer Septikämie durch *Klebsiella aerogenes* aufgrund einer vasookklusiven Krise. Diese gehen gehäuft mit Knochennekrosen und endogener Keimbesiedelung einher. Die Eradikation der Keime und die Frakturversorgung werden zu einer Herausforderung. Eine erfolgreiche Therapieoption sind wiederholte operative Eingriffe mit Segmenttransfer.

**Video online:**

Die Online-Version dieses Beitrags (10.1007/s00113-023-01334-9) enthält Videos von der Nachuntersuchung des Patienten 6 Monate nach der Osteosynthese.

## Abstract (en)

The vaso-occlusive crises of sickle cell disease are accompanied by bone necrosis, which favors endogenous bacterial colonization and thus osteomyelitis. This poses a major challenge for eradication and fracture management.

A 22-year-old patient with sickle cell disease sustained a multifragmentary, humeral shaft fracture. During surgical management, pus drained from the fracture site and further diagnostic work-up revealed osteomyelitis with evidence of *Klebsiella aerogenes*. Septicemia due to *Klebsiella aerogenes* had been treated 5 months prior to the accident, which occured because of a vaso-occlusive crisis. This is associated with clustered bone necrosis and endogenous germ colonization. Eradication of the germs and fracture care become a challenge. Repeated surgical procedures with segmental transfer can be a successful treatment option.

## Anamnese

Ein 22-jähriger Patient afrikanischer Abstammung stellte sich nach einer tätlichen Auseinandersetzung mit Verdrehtrauma des rechten Oberarms in der Notaufnahme vor. Vor dem Unfall habe der Patient keine Schmerzen im rechten Oberarm gehabt. Er ist homozygoter Träger des Sichelzellalleles. Die Krankheit wurde in seinem 3. Lebensjahr erstmalig symptomatisch in Form einer vasookklusiven Krise, welche sich in der Pubertät häuften und ca. 2‑mal jährlich auftraten. Seit dem 16. Lebensjahr erfolgt die intermittierende zytostatische Therapie mit Hydroxyurea und Austauschtransfusionen im 4‑wöchigen Rhythmus, um die schmerzhaften vasookklusiven Phasen zu verhindern. Weitere Nebenerkrankungen und Noxen sind nicht bekannt.

Fünf Monate vor der Fraktur war der Patient stationär aufgrund einer Septikämie. In Blutkulturen konnte *Klebsiella aerogenes* nachgewiesen werden. Ein Fokus der Spetikämie wurde nicht ausgemacht. Die Therapie erfolgte zunächst mit Piperacillin/Tazobactam und wurde dann auf Meropenem umgestellt.

## Befund

**Figure 1:**
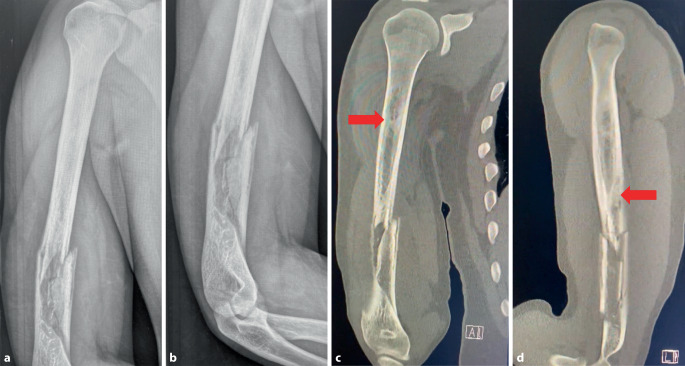

Der Patient beklagte Schmerzen im rechten Oberarm. Die Schulter und der Ellenbogen waren frei beweglich. Die Arme zeigten ein symmetrisches Muskelrelief. Die periphere Durchblutung, Motorik und Sensibilität waren intakt. Projektionsradiologisch zeigten sich eine mehrfragmentäre Humerusschaftfraktur sowie multiple diaphysäre Sklerosen. In der anschließenden Computertomographie (CT) wurden diese als Knocheninfarkte bei Sichelzellkrankheit gewertet (Abb. 1).

Die Leukozyten waren minimal auf 12,9 Tsd/ml erhöht, und das C‑reaktive Protein (CRP) lag mit < 4 mg/ml im Normalbereich. Die Körpertemperatur betrug 36,6 °C.

## Diagnose

Pathologische Humerusschaftfraktur (AO-12C).

## Therapie und Verlauf

**Figure 2:**
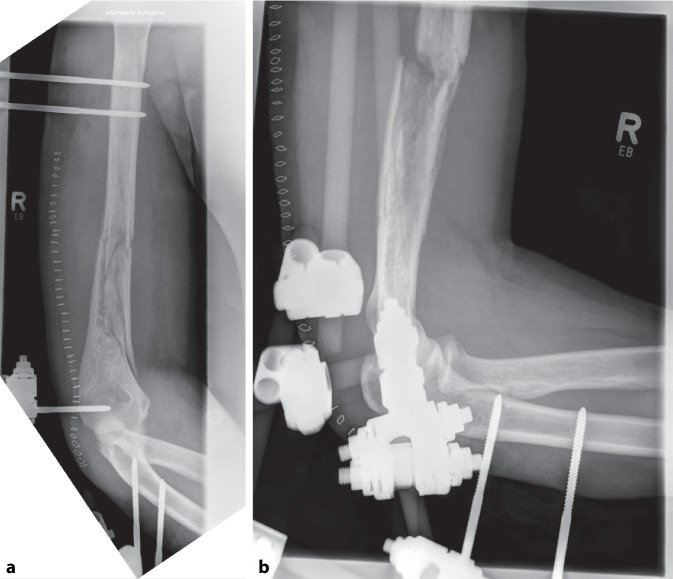

**Figure 3:**
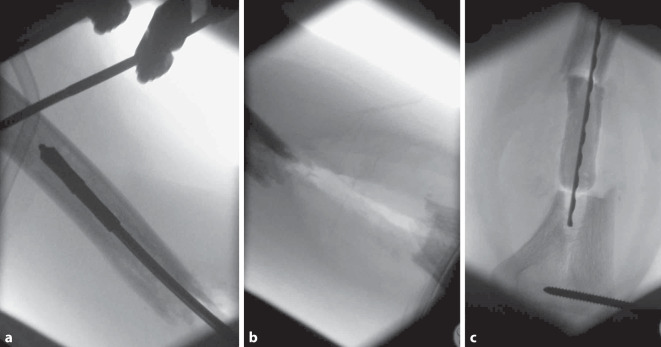

**Figure 4:**
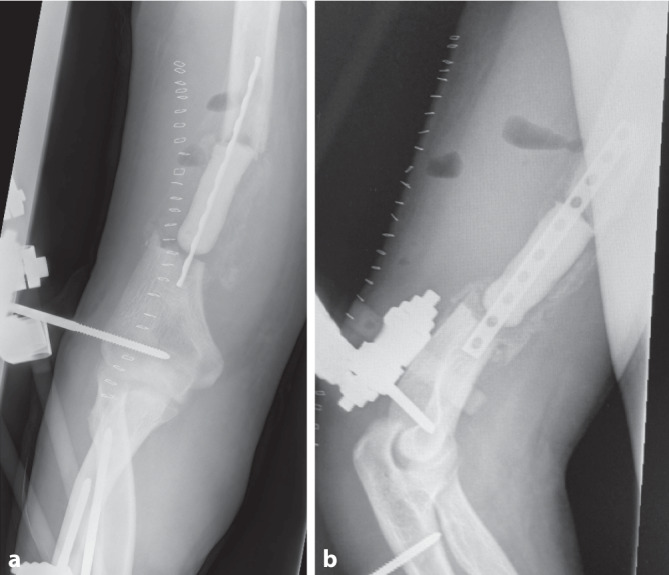

**Figure 5:**
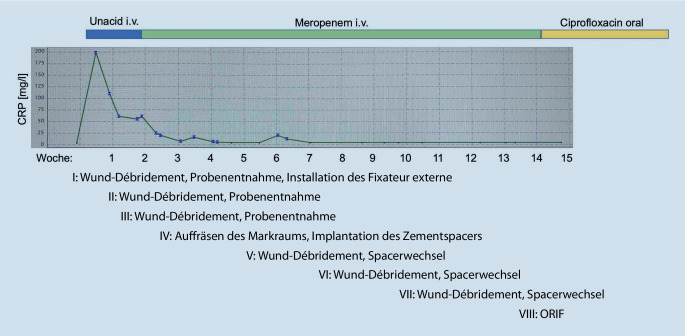

**Figure 6:**
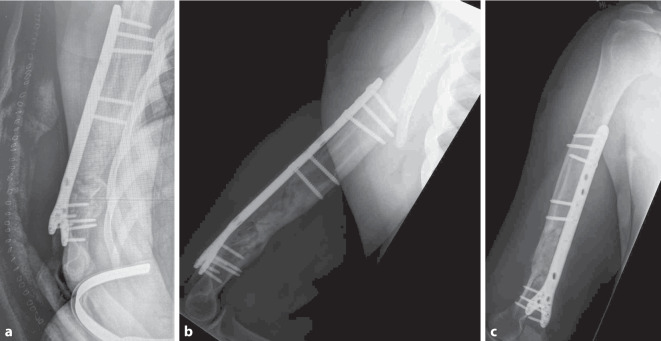

Nach Diagnosesicherung war noch am Unfalltag eine Plattenosteosynthese geplant. Intraoperativ entleerte sich jedoch reichlich Pus aus der Frakturzone. Das chirurgische Verfahren wurde gewechselt auf einen ellenbogengelenkübergreifenden Fixateur externe (Abb. 2). Es wurden 6 Proben aus dem Markraum bzw. dem ventralen Fragment entnommen, in 5 Proben konnte *Klebsiella aerogenes* isoliert werden. Direkt postoperativ wurde eine kalkulierte Antibiose mit Unacid i.v. begonnen. Die histologische Aufarbeitung der Proben bestätigte eine chronische Osteomyelitis. Es folgten 7 weitere Operationen mit ausgiebigem Débridement und Auffräsen des Markraums sowie Einlage eines Zementspacers (Abb. 3, 4). Dieser war mit Gentamicin und Vancomycin (COPAL G + V, Heraeus Medical, Wehrheim, Deutschland) beladen und musste mehrfach gewechselt werden, um den chronischen ossären Infekt zu beherrschen. Bei jeder Revisionsoperation wurde Material aus unterschiedlichen Regionen um die Fraktur gewonnen, insgesamt 35 Proben für die Mikrobiologie und 5 für die Pathologie. Nach der 4. Operation wurde *Staphylococcus warneri* als weiterer Erreger nachgewiesen. Die Antibiose wurde auf Meropenem eskaliert und insgesamt über einen Zeitraum von 13 Wochen gegeben (Abb. 5). Während des gesamten Zeitraums waren die laborchemischen Entzündungsparameter stets niedrig, und der Fixateur externe wurde bis zur endgültigen Osteosynthese in situ belassen. Bei fehlendem Keimnachweis zur letzten Revision vor 2 Wochen konnte die Rekonstruktion der Extremität angebahnt werden. Die 4 cm lange Defektstrecke am Humerus wurde mit autogener Beckenkammspongiosa überbrückt, sodass die Extremität nur minimal verkürzt werden musste (Abb. 6).

Die definitive Osteosynthese erfolgte mittels Y‑Platte („Rescue-Plate“, Fa. Königssee Implantate GmbH). Zur Nachbehandlung war der Ellenbogen für 2 Wochen im Oberarmgips ruhiggestellt. Anschließend waren eine freie Beübung ohne Gewicht sowie 4 Wochen Ciprofloxacin oral empfohlen.

In der Nachuntersuchung, 6 Monate nach der Osteosynthese, zeigt sich eine knöcherne Heilung des autogenen Spongiosatransplantats. Das Bewegungsausmaß in der rechten Schulter ist frei. Im Ellenbogengelenk ist eine Extension/Flexion von 0‑20-130° möglich, bei freier Pronation und Supination (Zusatzmaterial online: Videos 1 + 2).

## Diskussion

Die Sichelzellanämie ist eine Hämoglobinopathie, hervorgerufen durch eine Mutation der β‑Kette des Hämoglobins (HbS). Die Krankheit wird autosomal-rezessiv vererbt, und nur homozygote Träger entwickeln die Sichelzellkrankheit mit korpuskulärer, hämolytischer Anämie [1]. Die veränderte Rheologie der Erythrozyten führt zu einer Reduktion des Knochenmetabolismus mit Nekrosen und vasookklusiven Krisen. Evolutionär gesehen bietet das HbS einen Schutz vor einer Infektion mit *Plasmodium falciparum*. Heterozygote Träger von HbS haben hierdurch einen natürlichen Schutz vor Malaria ohne weitere Krankheitssymptome [2]. Epidemiologische Studien zeigten, dass sich das Vorkommen von den Malaria-Erregern mit der Prävalenz der Sichelzellkrankheit überschneidet [3]. Somit erklärt sich, weshalb in Afrika Schätzungen zufolge jährlich 200.000 Kinder als homozygote Träger des HbS geboren werden [1].

Die Sichelzellkrankheit ist insgesamt noch sehr selten in Deutschland. Kunz et al. gingen im Jahr 2020 von 2000 homozygoten Trägen in Deutschland aus [4]. Daher dürfte diese Auswirkung der Krankheit auf den Knochen noch weniger im klinischen Bewusstsein präsent sein. Die veränderte Rheologie und die verminderte Sauerstoffversorgung im heilenden Knochen machen jede Osteosynthese zur Herausforderung [5]. Der pathologische Knochenstoffwechsel erhöht das Risiko für eine Infektion immens, und das Keimspektrum unterscheidet sich zu Osteomyelitiden in der Normalbevölkerung [6]. In dem beschriebenen Fall war 5 Monate vor der Fraktur eine Septikämie unklarer Ursache aufgetreten. Eine spezifische Empfehlung zur kalkulierten Antibiose von Osteomyelitis bei Sichelzellkranken existiert nicht [7].

Zuweilen können native Röntgen- oder CT-Bilder missinterpretiert werden, da die pathogenetischen „Popcorn-artigen“ Veränderungen im Knochen als inaktive Nekrosen abgetan werden. Differenzialdiagnostisch sollte eine aktive Osteomyelitis mittels Kontrastmittel-MRT ausgeschlossen werden [8].

In Anbetracht der fortschreitenden Globalisierung wird voraussichtlich auch die Anzahl Sichelzellkranker in Deutschland zunehmen [9]. Um der veränderten Knochenbiologie gerecht werden zu können, verlangen diese pathologischen Frakturen ein besonderes Augenmerk, sowohl in der Diagnostik wie der operativen Versorgung.

## Fazit für die Praxis

- Weltweite Migrationsbewegungen können mit einer Prävalenzsteigerung der Sichelzellkrankheit in Deutschland einhergehen.
- Der sklerotische Knochen bei Sichelzellkranken heilt verzögert.
- Nach Osteosynthese besteht eine erhöhte Gefahr für eine Superinfektion der Knochennekrosen.
- Um eine Osteomyelitis mit untypischen Erregern zu erkennen, sollten Proben zur mikrobiologischen, molekulargenetischen und histologischen Aufarbeitung entnommen werden.
- Die Indikation für einen Fixateur externe ist großzügig zu stellen.

### Supplementary Information





